# The Law of Hospitals

**Published:** 1908-06-13

**Authors:** 


### THE LAW OF HOSPITALS.

The Law of Hospitals, Infirmaries, Dispensaries, and other Kindred Institutions, whether Voluntary or Rate-supported. By Arthur Turnour Murray, Barrister-at-law. Pp. 287. (London: John Murray. Price 10s. 6d. net cash.)

It must surely have struck those connected with hospitals, and more especially voluntary hospitals, that no competent lawyer should have set himself the task of collecting all the scattered branches of the law which appertain thereto. Yet, if we set aside the legal handbook for the use of hospital authorities which was compiled some years ago by the then Mr. L. S. Bristowe, the fact remains that in this country at any rate no very definite attempt had been made to supply the deficiency.

Mr. Turnour Murray has now come forward with a treatise upon this important and technical branch of the law, arranged in such a form as to indicate, briefly it may be, but none the less accurately, all the various points connected with the administration and liabilities of the various types of institution which minister to the bodily wants of the sick in England and Wales. The book is arranged in the form of an abridgement, and covers a variety of subjects, commencing with "Accounts" and "Ambulances," touching in alphabetical order such diverse topics as "Gifts to Hospitals," "Insurance," "Medical Schools," "Naval and Military Hospitals," "Officers and Servants," "Poor-law Infirmaries," and "Rates, Taxes, and Duties," and concluding with "Vivisection" and "Voluntary Hospitals." There is also a careful and accurate index. It will thus be seen that many classes and types of hospital officers and persons interested can now be supplied at first hand with the latest law on subjects with which they are brought into continual contact, whilst the medical staff will find a fund of useful information in connection with their professional obligations. This work is no bald statement of a variety of legal propositions compiled without reference to authority or context and amassed by dint of copious extracts from the text-books peculiar to each subject; on the contrary, the author has evidently spared no pains to bring together, illustrate, and describe in terse and lucid language the legal authorities and dicta upon which his work is based. Nearly 250 reported cases, both English and American, are cited, and, in dealing with the rate-supported hospital, the somewhat intricate provisions of the Public Health Acts and of the statutes relating to infectious diseases and isolation hospitals are quoted and explained.

Mr. Murray has evidently kept the utilitarian possibilities of his work clearly in mind, for he finds room to devote a very able chapter to the law relating to meetings—a fact which should enhance the value of the book as a work of reference for the board-room table. It is impossible, within the space available, to do justice to this valuable addition to the standard literature of a legal-institutional character; but we can heartily recommend it to the governors, secretaries, and staffs of all hospitals in this country, whatever their nature may be, and we venture to express the opinion that both branches of the legal profession will be the gainers, owing to the fact that Mr. Turnour Murray has supplied a valuable and authoritative text-book upon a difficult and intricate branch of the law.

